# Characterisation of the allergic phenotype resulting from a shortened HDM challenge protocol in mice

**DOI:** 10.1186/1476-9255-10-S1-P6

**Published:** 2013-08-14

**Authors:** Sorif Uddin, Joanne Morley, Sara Hughes, Edith Hessel

**Affiliations:** 1 GlaxoSmithKline, Stevenage, UK

## Background

Most mouse models of house dust mite (HDM) challenge-induced allergic pulmonary inflammation utilise long challenge periods usually via the intranasal route that can typically last for up to 9 weeks. While these extended protocols are useful for investigating features of lung remodelling, the allergic phenotype, consisting of the cell and cytokine/chemokine network, usually develops early on. We describe here the phenotype of the allergic process in a truncated 3 week protocol.

## Materials and methods

Female BALB/c mice were intra-nasally challenged once a day for 5 days a week for a 3 week period with HDM extract (25µg) in 50µl saline. Mice were sacrificed 4 hours after the last HDM challenge on the third week and bronchoalveolar lavage levels of; cells, chemokines, cytokines and serum IgE were quantified. Airways hyper-responsiveness (AHR) by whole body plethysmography to increasing concentrations of 5-Hydroxytryptamine was ascertained 24 hours prior to sacrificing animals. Separate groups of mice were also challenged intra-nasally with anti-CD3e (1µg in 50µl saline) at 24, 48 or 72 hours post 1, 2 or 3 weeks of HDM exposure. The resulting cytokine levels in bronchoalveolar lavage were ascertained 4 hours after anti-CD3e challenge. This was to test the ability of resident T cells to generate a cytokine response.

## Results

Apart from macrophages, all cell types investigated were elevated above background levels 4 hours after the last HDM challenge. The presence of CD4+ cells in lung lavage was associated with an increase in MDC and TARC levels. Serum total IgE levels were also elevated with a small, above background increase in HDM-specific IgE. HDM challenged animals exhibited an increased AHR compared to saline challenged animals. Anti-CD3e challenge elicited a robust cytokine signal at the 48 hour time-point on the third week of HDM challenge.

## Conclusions

The model described here is a refinement of HDM protocols described in the literature that can last for months. This modified protocol allows for the investigation of allergic mechanisms at an earlier time-point resulting in less cost to animals and a quicker data turnaround time. This approach may be more conducive to the optimisation of novel therapeutic agents.

